# Stochasticity in host-parasitoid models informs mechanisms regulating population dynamics

**DOI:** 10.1038/s41598-021-96212-y

**Published:** 2021-08-18

**Authors:** Abhyudai Singh

**Affiliations:** 1 Department of Electrical and Computer Engineering, University of Delaware, Newark, DE 19713 USA; 2 Department of Biomedical Engineering, University of Delaware, Newark, DE 19713 USA; 3 Department of Mathematical Sciences, University of Delaware, Newark, DE 19713 USA; 4 Center for Bioinformatics and Computational Biology, University of Delaware, Newark, DE 19713 USA

**Keywords:** Ecological modelling, Population dynamics, Theoretical ecology, Applied mathematics, Entomology

## Abstract

Population dynamics of host-parasitoid interactions have been traditionally studied using a discrete-time formalism starting from the classical work of Nicholson and Bailey. It is well known that differences in parasitism risk among individual hosts can stabilize the otherwise unstable equilibrium of the Nicholson-Bailey model. Here, we consider a stochastic formulation of these discrete-time models, where the host reproduction is a random variable that varies from year to year and drives fluctuations in population densities. Interestingly, our analysis reveals that there exists an optimal level of heterogeneity in parasitism risk that minimizes the extent of fluctuations in the host population density. Intuitively, low variation in parasitism risk drives large fluctuations in the host population density as the system is on the edge of stability. In contrast, high variation in parasitism risk makes the host equilibrium sensitive to the host reproduction rate, also leading to large fluctuations in the population density. Further results show that the correlation between the adult host and parasitoid densities is high for the same year, and gradually decays to zero as one considers cross-species correlations across different years. We next consider an alternative mechanism of stabilizing host-parasitoid population dynamics based on a Type III functional response, where the parasitoid attack rate accelerates with increasing host density. Intriguingly, this nonlinear functional response makes qualitatively different correlation signatures than those seen with heterogeneity in parasitism risk. In particular, a Type III functional response leads to uncorrelated adult and parasitoid densities in the same year, but high cross-species correlation across successive years. In summary, these results argue that the cross-correlation function between population densities contains signatures for uncovering mechanisms that stabilize consumer-resource population dynamics.

## Introduction

Fluctuations in population densities are an inherent feature of all ecological systems. While in some cases these fluctuations can be attributed to seasonal variations or chaotic dynamics^1^, demographic and environmental stochasticity have been shown to be key drivers of population density fluctuation^2–4^. Demographic stochasticity is related to the random birth/death of individuals that become particularly important for small population sizes. In contrast, environmental stochasticity reflects random changes in environmental conditions that are often modeled by modifying ecological parameters as random processes. Both forms of stochasticity have been well studied in the continuous-time framework of modeling predator-prey dynamics, where stochasticity can be added through Brownian noise terms leading to several insights on noise-driven population extinctions^5–11^. In the discrete-time formalism of modeling population dynamics such effects have been studied in single-species models^12,13^, but systematic investigation of stochasticity is missing in complex two-species models of consumer-resource dynamics. In this contribution, we leverage the rich body of work on deterministic models of host-parasitoid interactions in the discrete-time setting to study the impacts of random yearly variations in host reproduction. We use a combination of analytical tools and stochastic simulations to understand how the extent of environmental stochasticity affects population density fluctuations, and how these effects differ across models with different parasitoid search/attack mechanisms. We start by briefly reviewing deterministic host-parasitoid models and then later turn to the analysis of stochastic counterparts of these models that include environmental stochasticity.

Population dynamics of host-parasitoid interactions is typically formulated as a discrete-time model

1a $$\begin{aligned} H_{t+1}= & {} RH_tf(H_t,P_t) \end{aligned}$$

1b $$\begin{aligned} P_{t+1}= & {} kRH_t[1-f(H_t,P_t)] \end{aligned}$$

where $H_t$ and $P_t$ are the adult host, and the adult parasitoid densities, respectively, in year *t*^14–20^. Without loss of any generality, we assume that the host becomes vulnerable to parasitoid attacks in the larval stage. If $R>1$ denotes the number of viable eggs produced by each adult host, then $RH_t$ is the host larval density exposed to parasitoid attacks. Adult (female) parasitoids search and attack host larvae with the density-dependent function $f(H_t,P_t)<1$ representing the *escape response*, i.e., the fraction of host larvae escaping parasitism. Thus, $RH_tf(H_t,P_t)$ is the total larval density escaping parasitism that metamorphosize as adults the following year. Finally, $RH_t[1-f(H_t,P_t)]$ is the density of parasitized larvae, where the juvenile parasitoid develops at the host’s expense by using it as a food source that ultimately results in host death. The juvenile parasitoids pupate and emerge as adult parasitoids the following year. Considering that each parasitized larvae gives rise to *k* adult female parasitoids in the next generation, it results in the update function (1b) for the adult parasitoid density.

Perhaps the simplest formulation of (1) is the classical Nicholson-Bailey model

2a $$\begin{aligned} H_{t+1}= & {} RH_t\exp (-cP_t) \end{aligned}$$

2b $$\begin{aligned} P_{t+1}= & {} kRH_t[1-\exp (-cP_t)] \end{aligned}$$

with a parasitoid-dependent escape response $\exp (-cP_t)$, where $c>0$ represents the rate at which parasitoids attack and parasitize host larvae^21^. The Nicholson–Bailey model is characterized by diverging oscillations in population densities resulting in an unstable population dynamics^21^. Much work has identified two orthogonal mechanisms by which stability can arise in these discrete-time models:

- The first mechanism is when the escape response $f(P_t)$ only depends on the parasitoid density, and then the non-trivial host-parasitoid equilibrium is stable, if and only, if, the equilibrium adult host density is an *increasing* function of the host reproduction rate *R*^22^. This type of stability arises through several related processes, such as, a fraction of the host population being in a refuge (i.e., protected from parasitoid attacks)^16,23^, large host-to-host difference in parasitism risk^22,24–26^, parasitoid interference^27–29^, and aggregation in parasitoid attacks^30–32^.
- The second mechanism is a Type III functional response where the parasitoid attack rate accelerates sufficiently rapidly with increasing host density^33,34^. Here the escape response *f* depends on both the host and parasitoid densities, and interestingly, in this case stability leads to the adult host equilibrium density being a *decreasing* function of the host reproduction rate *R*^35^. Parasitoids have tremendous potential for biological control of pest species^36–39^, and a Type III functional response has been shown to suppress the host density to arbitrary low levels while maintaining system stability^35^.

In this contribution, we consider annual variations in host reproduction that drive fluctuations in the host/parasitoid population densities^2^. These random fluctuations are investigated in the context of two alternative stabilizing mechanisms: variation in parasitism risk across hosts and a Type III functional response. Our analysis develops analytical formulas that quantifies the extent of variations in population densities as a function of ecological parameters and shows that harnessing the statistics of population fluctuations can be a vital tool for discriminating between stability mechanisms and characterizing host-parasitoid interactions. We start by incorporating host-to-host differences in parasitism risk in the Nicholson-Bailey model (2).

## Variation in parasitism risk

The Nicholson-Bailey model assumes that all hosts are identical in terms of their vulnerability to parasitism. Perhaps a more realistic scenario is individual hosts differing in their risk of parasitism due to genetic factors, spatial heterogeneities, or are exposed to parasitoids for different durations, and at different times^40–43^. In essence, the attack rate *c* in (2) can be interpreted as “parasitism risk”, and by transforming it into a random variable we obtain

3a $$\begin{aligned} H_{t+1}= & {} RH_tf(P_t), \ \ f(P_t)=\int _{x=0}^\infty p(x)\exp (-xP_t) \end{aligned}$$

3b $$\begin{aligned} P_{t+1}= & {} k RH_t\left[ 1-f(P_t)\right] \end{aligned}$$

where *p*(*x*) is the distribution of risk across hosts^22^. A key assumption in this formulation is that risk is independent of the local host density, if hosts are non-uniformly distributed in space. Assuming *p*(*x*) follows a gamma distribution with mean $\bar{c}$ and coefficient of variation *CV* yields the escape response

4 $$\begin{aligned} f(P_t)=\int _{x=0}^\infty p(x)\exp (-xP_t)=\frac{1}{\left( 1+\bar{c}CV^2P_t\right) ^\frac{1}{CV^2}}. \end{aligned}$$

The non-trivial fixed point of the model (3)-(4) is given by

5 $$\begin{aligned} P^*=\frac{R^{CV^2}-1}{\bar{c}CV^2}, \quad H^*=\frac{R^{CV^2}-1}{k\bar{c}CV^2(R-1)}, \end{aligned}$$

where $P^*$ and $H^*$ denote the parasitoid and host equilibrium densities, respectively. Prior analysis has shown that when the escape response$f(P_t)$ only depends on the parasitoid density, and then the non-trivial host-parasitoid equilibrium is stable, if and only, if,

6 $$\begin{aligned} \frac{dH^*}{dR}>0 \end{aligned}$$

^22^. In recent work we have generalized this condition to arbitrary escape responses that can depend on both host/parasitoid densities, and analysis shows that stability occurs more often when *f* is a decreasing function of the host density, rather than an increasing function^44^. Applying the condition (6) to (5) straightforwardly leads to a classical result - $CV>1$ stabilizes the population dynamics irrespective of model parameters *R* and $\bar{c}$^24,25,32^. The stabilizing risk distribution implies that a majority of hosts are at low risk, and stability arises from parasitoid attacks being skewed towards a small fraction of high-risk individuals. This stability criterion motivated several studies investigating spatial patterns of parasitism in the field, and many data sets were found to be consistent with $CV>1$^26^. Recent work in this direction has relaxed the assumption of a gamma-distributed risk. It turns out that if $R\approx 1$, then $CV>1$ is the necessary and sufficient condition for stability irrespective of what form *p*(*x*) takes. However, for $R \gg 1$, stability requires a skewed risk distribution with the modal risk being zero (as in the gamma distribution for $CV>1$)^22^.

## Incorporating yearly fluctuations in host reproduction

Working with model (3)-(4) that considers a Gamma distributed risk, we incorporate random fluctuations in host reproduction by replacing *R* with an independent and identically distributed random variable $R_t$ with mean *R* and variance $\sigma ^2_R$. Considering small perturbations $h_t$, $p_t$ around the equilibrium densities (5)

7 $$\begin{aligned} h_t:=H_t-H^*, \ \ \ p_t:=P_t-P^*, \end{aligned}$$

model (3)-(4) can be written as the following noise-driven linear discrete-time system

8 $$\begin{aligned} \left[ \begin{array}{c} h_{t+1} \\ p_{t+1} \end{array} \right] =A\left[ \begin{array}{c} h_{t} \\ p_{t} \end{array} \right] +Br_t, \quad r_t:=R_t-R, \end{aligned}$$

where the entries of the Jacobian matrix *A* are given by

9 $$\begin{aligned} A=\left[ \begin{array}{lr} 1 &{} R H^* \frac{d f(P_t)}{d P_t}|_{P_t=P^*} \\ k(R-1) &{} - k R H^* \frac{d f(P_t)}{d P_t}|_{P_t=P^*} \end{array} \right] . \end{aligned}$$

Here $\frac{d f(P_t)}{d P_t}|_{P_t=P^*}$ represents the derivative of the escape response with respect to $P_t$ evaluated at the equilibrium point, and assuming a stable host-parasitoid equilibrium, all eigenvalues of *A* are inside the unit circle^45,46^. The matrix *B* in (8) is given by

10 $$\begin{aligned} B=\left[ \begin{array}{c} \frac{H^*}{R} \\ k H^*\left( 1- \frac{1}{R} \right) \end{array} \right] \end{aligned}$$

and characterizes the random forcing of the system by the zero-mean random variable $r_t$.

**Figure 1:**
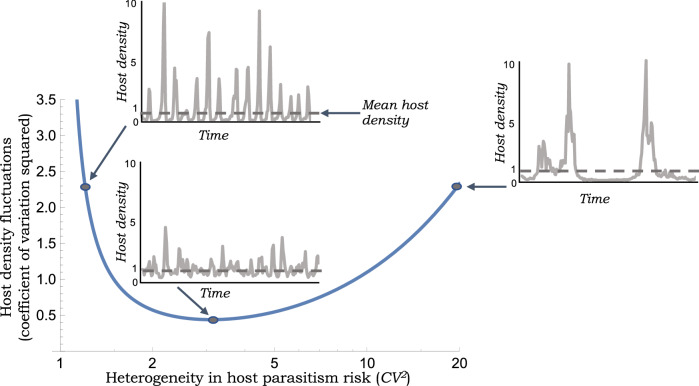
Extent of fluctuations in the host population density are minimized at an intermediate level of heterogeneity in parasitism risk. The steady-state coefficient of variation squared of the host population density as predicted by (14) and (16) is plotted as a function of the heterogeneity in parasitism risk *CV*. Three examples of host density fluctuations are generated by performing stochastic simulations of model (3)-(4) for different values of *CV* assuming $\bar{c}=1$, $R=2$ and $\sigma ^2_R=1$. All time series are normalized to have a mean value of one.

Let

11 $$\begin{aligned} C=\lim _{t \rightarrow \infty }\left[ \begin{array}{lr} \langle h_t h_t\rangle &{} \langle h_t p_t\rangle \\ \langle h_t p_t\rangle &{} \langle p_t p_t\rangle \end{array} \right] \end{aligned}$$

denote the steady-state covariance matrix, where $\langle \ \rangle$ represented the expected value operation. Then the covariance matrix is the unique solution to the Lyapunov equation

12 $$\begin{aligned} A C A^T+B B^T \sigma ^2_R=C, \quad \sigma ^2_R:= \langle r_t r_t\rangle \end{aligned}$$

^47^. For a two-dimensional system, the Lyapunov equation can be solved analytically to yield

**Figure 2:**
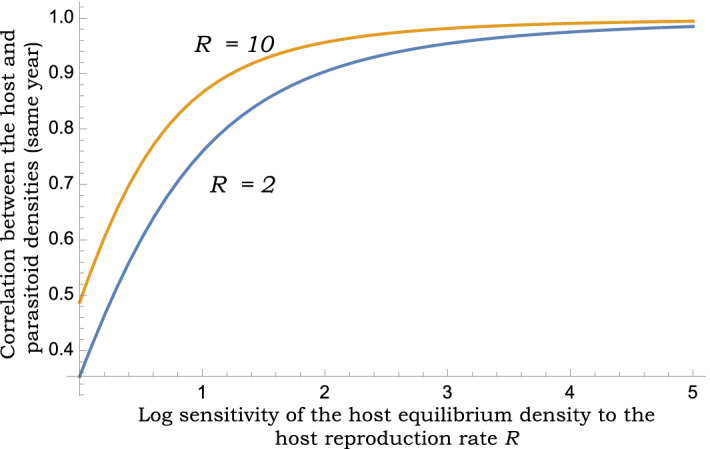
Randomness in host reproduction induces strong positive correlations between host-parasitoid densities for model (3)–(4) that incorporates heterogeneity in parasitism risk. Predicted Pearson correlation coefficient between the host and parasitoid densities for $R=2$ and $R=10$ as a function of $H_R$ as given in (14).

13 $$\begin{aligned} CV^2_H := \lim _{t \rightarrow \infty } \frac{\langle h_t h_t\rangle }{{H^*}^2}=\frac{H_R^3-2H_R(1+H_R+H_R^2)R+(2+H_R(2+H_R(2+H_R)))R^2}{H_R(R-1)R^2(1-2H_R+3R+2H_RR)}\sigma ^2_R \end{aligned}$$

where $CV^2_H$ is the steady-state coefficient of variation squared of the host population density and

14 $$\begin{aligned} H_R:= \frac{R}{H^*} \frac{dH^*}{dR}=CV^2+\frac{CV^2}{R^{CV^2}-1}-\frac{R}{R-1} \end{aligned}$$

in the dimensionless log sensitivity of the host equilibrium density to *R*. Using (5), it can be seen that $H_R$ is monotonically related to the heterogeneity in the Gamma distributed risk as quantified by its coefficient of variation *CV*. In particular, higher levels of *CV* increase $H_R$ making $H^*$ more sensitive to *R*. Recall from (6) that the stability of the deterministic discrete-time system implies $H_R>0$. Interestingly, a close inspection of (13) reveals

15 $$\begin{aligned} \lim _{H_R \rightarrow 0} CV^2_H =\infty \quad \mathrm{and} \quad \lim _{H_R \rightarrow \infty } CV^2_H =\infty \end{aligned}$$

implying $CV^2_H$ is minimized at an intermediate value of $H_R$. For example, where $R=2$, then (13) reduces to

16 $$\begin{aligned} CV^2_H =\frac{8+H_R(2+H_R)^2}{4H_R(7+2H_R)}\sigma ^2_R \end{aligned}$$

which is minimized where $H_R \approx 1.51$. From (14), this corresponds to host density fluctuations being minimal when $CV \approx 1.76$ (Fig. 1). The magnitude of fluctuations in the parasitoid population density also follows a similar U-shape profile with increasing *CV*. Solving the Lyapunov equation (12) leads to the following Pearson correlation coefficient between the host and parasitoid densities (assuming $R=2$)

17 $$\begin{aligned} \rho _{H,P}: =\lim _{t \rightarrow \infty }\frac{ \langle h_t p_t\rangle }{\sqrt{ \langle h_t h_t\rangle }\sqrt{ \langle p_t p_t\rangle }} =\frac{2+H_R(4+H_R)}{\sqrt{4+H_R}\sqrt{8+H_R(2+H_R)^2}} \end{aligned}$$

that predicts a moderate to strong correlation depending on $H_R$ (Fig. 2). This strong correlation can be intuitively explained by the fact that both the host and parasitoid equilibrium densities (5) are monotonically increasing functions of *R*. Interestingly, our analysis reveals that the extent of fluctuations in population densities (as determined by the coefficient of variation), and the correlation coefficient $\rho _{H,P}$ are completely independent of *k*, the number of parasitoids emerging from a single larvae.

## Stability arising through a type III functional response

We next focus our attention on another stabilizing mechanism based on density-dependence in the parasitoid attack rate. In our prior work, we have considered a Type III parasitoid functional response, where the attack rate $cL^m$ accelerates with increasing host larvae density *L* for some positive constant *c* and exponent *m*. Here, *L* denotes the non-parasitized larval density that decreases overtime during the vulnerable stage leading to a variable attack rate. To capture such effects of populations changing continuously within the larval stage of each year, a semi-discrete or hybrid formalism has been proposed to mechanistically formulate the corresponding discrete-time model. This semi-discrete approach relies on solving a continuous-time differential equation describing population interaction during the host’s vulnerable stage to derive update functions connecting population densities across consecutive years^33,48–51^. For an attack rate $cL^m$ this leads to the model (1) with escape response

18 $$\begin{aligned} f(RH_t,P_t)=\frac{1}{\left( 1+cm\left( RH_t\right) ^mP_t\right) ^\frac{1}{m}} \end{aligned}$$

that depends on both host and parasitoid population densities^33^. It turns out that the model’s unique non-trivial fixed point

19 $$\begin{aligned} H^*=\left( \frac{R^m-1}{kcmR^m(R-1)}\right) ^\frac{1}{1+m}, \quad P^*=k(R-1)H^*, \end{aligned}$$

is stable iff $m>1$, and $m=1$ results in a neutrally stable equilibrium where populations oscillate with a period of $2\pi /\arctan (\sqrt{R^2-1})$^33^. Interestingly, in contrast to (6), here $H^*$ is a decreasing function of *R*, while $P^*$ is an increasing function of *R*. It is important to point out that a phenomenological approach of incorporating a Type III functional response by simply substituting *c* in the Nicholson-bailey model (2) with $c(RH_t)^m$ (i.e., the parasitoid attack rate is set by the initial larval density $RH_t$ and remains fixed through the larval stage) leads to an unstable population equilibrium for all $m\ge 0$^52,53^.

As done in the previous section, considering stochastic fluctuations in the host reproduction rate in the model defined by (1) and (18) yields the Lyapunov equation (12) with

**Figure 3:**
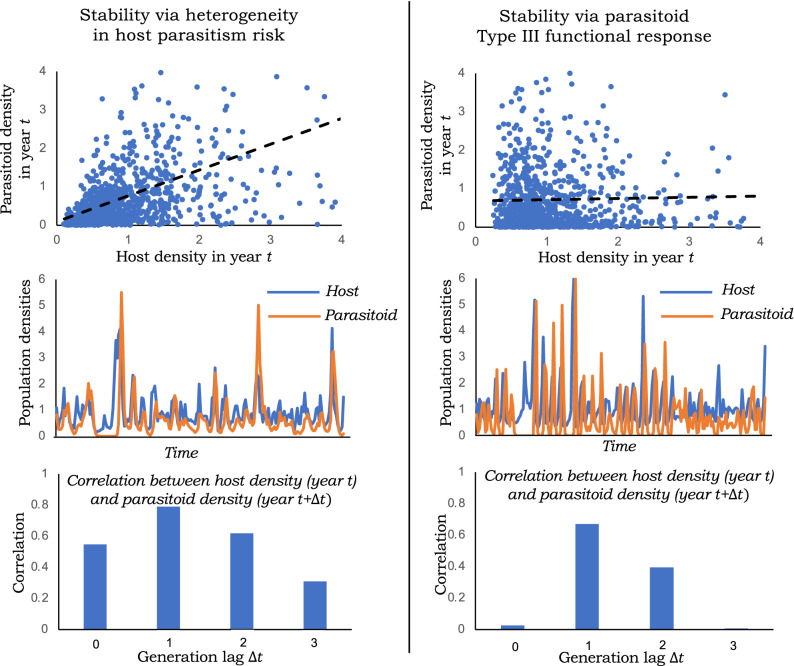
Different stabilizing mechanisms of host-parasitoid population dynamics can be discriminated from the cross-correlation function profile. *Left*: Scatter plot of $H_t$ and $P_{t}$ from a stochastic simulation of model (3)-(4) for $CV=2$, $\bar{c}=1$, $R=2$, $\sigma ^2_R=1$ along with the time-series of host/parasitoid population densities. Heterogeneity in parasitism risk results in strong correlation between $H_t$ and $P_{t}$, that shows a modest increase followed by decay to zero with increasing generation lag $\Delta t$ for correlations between $H_t$ and $P_{t+\Delta t}$. *Right*: Scatter plot of $H_t$ and $P_{t}$ from a stochastic simulation of model (1) and (18) for $m=2$, $\bar{c}=1$, $R=2$, $\sigma ^2_R=1$ reveals uncorrelated fluctuations in host/parasitoid densities for a Type III functional response. As can be seen in the simulated time-series and the cross-correlation function, $h_t$ and $p_{t+1}$ show a strong positive correlation that decays back to zero with increasing generation lag $\Delta t$.

20 $$\begin{aligned} A&=\left[ \begin{array}{lr} 1+R H^* \frac{\partial f(RH_t,P_t)}{\partial H_t}|_{H_t=H^*,P_t=P^*} &{} R H^* \frac{\partial f(RH_t,P_t)}{\partial P_t}|_{H_t=H^*,P_t=P^*} \\ k (R-1)-k R H^* \frac{\partial f(RH_t,P_t)}{\partial H_t}|_{H_t=H^*,P_t=P^*} &{} - k R H^* \frac{\partial f(RH_t,P_t)}{\partial P_t}|_{H_t=H^*,P_t=P^*} \end{array} \right] \end{aligned}$$

21 $$\begin{aligned} B&=\left[ \begin{array}{c} \frac{H^*}{R} +R H^* \frac{\partial f(RH_t,P_t)}{\partial R}|_{H_t=H^*,P_t=P^*} \\ k H^*\left( 1- \frac{1}{R} \right) -k R H^* \frac{\partial f(RH_t,P_t)}{\partial R}|_{H_t=H^*,P_t=P^*} \end{array} \right] . \end{aligned}$$

Solving the Lyapunov equation reveals that in this case the extent of fluctuations in host/parasitoid densities monotonically decreases to zero with increasing *m*. For example, for $R=2$, $CV^2_H \approx 0.32 \sigma ^2_R$ when $m=2$ and $CV^2_H \approx 0.13 \sigma ^2_R$ when $m=3$. This makes sense as increasing *m* not only increases system stability (i.e., faster return to equilibrium in response to perturbations) but also makes the host equilibrium less sensitive to *R* with

22 $$\begin{aligned} H_R:= \frac{R}{H^*} \frac{dH^*}{dR}=\frac{\frac{m}{R^m-1}-\frac{R}{R-1}}{1+m}<0, \quad \lim _{m \rightarrow \infty } H_R =0. \end{aligned}$$

We also obtain the following analytical expression for the cross-species Pearson correlation (assuming $R=2$)

23 $$\begin{aligned} \rho _{H,P} =\frac{2^m m^2-(2 (-1 + 2^m)^2 )}{ \sqrt{-2 + 2^m (2 + m)} \sqrt{ 8 (-1 + 2^m)^3 + m (4 (-1 + 2^m)^3 + (2 + 2^m (-2 + m)) m)}} \end{aligned}$$

that is predicted to be $\rho _{H,P} \approx -0.01$ when $m=2$ and $\rho _{H,P} \approx -0.03$ when $m=3$. Such uncorrelated fluctuations in host/parasitoid densities in response to random perturbations in *R* is reflective of $H^*$ and $P^*$ in (19) being a decreasing and increasing function of *R*, respectively. Intriguingly, if one quantifies the cross-correlation function across different years, i.e., the correlation between $H_t$ and $P_{t+\Delta t}$ where $\Delta t$ is the generation lag of the host with respect to the parasitoid, then one sees a sharp jump to positive cross-species correlation between $H_t$ and $P_{t+1}$ which then again goes back to zero with larger generation lags (Fig. 3). Hence, a Type III functional response is characterized by uncorrelated same-year fluctuations in population densities that exhibit a non-monotonic cross-correlation function profile. In contrast to these results, heterogeneity in parasitism risk leads to strong same-year correlations that gradually decay to zero with increasing $\Delta t$ (Figs. 2 and 3).

## Discussion

The interaction between a consumer (such as, a parasitoid) and a resource (such as, a host) forms a core motif in ecological food webs. Arthropod host-parasitoid interactions constitute an important class of consumer-resource systems with tremendous potential in biological control of pest population densities by using parasitoids as a natural enemy against pest insect species^17,36–39,54^. Discrete-time formalism is a tradition in modeling of host-parasitoid interaction starting from the seminal work of Nicholson and Bailey close to a century ago, and this framework is partly motivated by the univoltine life histories of insects living in the temperate regions of the world. The fact that the simplest Nicholson-Bailey model leads to an unstable interaction with diverging cycles of population densities fueled a rich body of theoretical/experimental work understanding the impact of diverse ecological processes on host-parasitoid population dynamics^16^.

One mechanism known to stabilize the host-parasitoid interaction is variation in parasitism risk across individual hosts with $CV > 1$ stabilizing the model equilibrium^22,24–26,43,55^, where *CV* is the coefficient of variation of the distribution of risk. Interestingly, parasitism field patterns for differed host-parasitoid systems were found to satisfy this stability criterion^26^. While much prior analysis has relied on deterministic models, a key novelty of this work is to consider annual random fluctuations in the parameter *R*: the number of viable eggs laid per adult host that become adult hosts next year. For the stochastic model, we developed closed-form expressions for the extent of fluctuations and correlations in population densities. An intriguing result from our analysis is that increasing *CV* beyond a critical point enhances population density fluctuation (as quantified by the coefficient of variation of population density), and hence can be destabilizing in the stochastic formulation (Fig. 1). This result can be intuitively understood in terms of (14) where the host population density becomes more sensitive to *R* at larger values of *CV*. Thus, low variation in parasitism risk drives large density fluctuations as the system is close to the instability boundary. In contrast, high variation in parasitism risk also leads to large fluctuations due to the enhanced sensitivity of the host equilibrium to the host reproduction rate. Another vital observation is that the host-parasitoid population densities are strongly correlated within the same year (Figs. 2& 3), and this is expected given that the equilibrium population densities (5) are both increasing functions of *R*. The cross-correlation function in Fig. 3 starts with a high same-year correlation, increasing slightly for a one-year lag, and then decreases to zero with increasing time lags. For large values of *CV* the cross-correlation function starts even higher, and then monotonically decreases to zero without showing the minor peak for a one-year lag.

A further analysis of (13) shows that the optimal value of *CV* that minimizes host density fluctuations in not very sensitive to the mean value *R*. Recall from Fig. 1, that for $R=2$ the minimum was achieved at $CV \approx 1.76$. We find the minimum to occur $CV \approx 1.56$ for $R=2$ and $CV \approx 1.51$ for $R=10$. How do these model predicted values compare to experimentally observed *CV* values? Using data on the host *Prokelisia marginata* and its parasitoid *Anagrus delicatus* from^30^, our prior analysis in^22^ had found that the host parasitism risk was independent of local host density across patches consistent with our model assumption. Furthermore, we had estimated a *CV* value of 1.31 with a $95\%$ confidence interval of (1.23, 1, 4) (see caption of Fig. 2 in^22^) that is in the same ballpark as predicted by the model to mitigate random fluctuations in host reproduction. To experimentally test the $CV^2>1$ rule needed for stability^26^, analyzed 34 published datasets and found 9 to satisfy the rule with *CV* values ranging between 1.2 to 2.7 (the third column in Table 3 of^26^ list the $CV^2$ value). These studies suggest that natural system don’t seem to exhibit large values of *CV* which is strongly stabilizing in the deterministic framework, but amplifies density fluctuations in the stochastic framework (Fig. 1).

Finally, we consider an alternative stabilizing mechanism based on a Type III functional response, where the parasitoid’s attack rate accelerates with increasing host density. While prior work had found such an accelerating response destabilizing in the discrete-time formulation, recent work using a semi-discrete approach has found them to be stabilizing similar to the continuous-time framework of Lotka-Volterra^16,33^. This discrepancy arises from the attack rate being phenomenologically set by the initial host density and not being allowed to vary continuously within the season as in the semi-discrete approach. In the presence of environmental stochasticity in *R*, a Type III functional response suppresses density fluctuations with the magnitude of fluctuations decreasing with increasing acceleration towards the host density. In contrast to variation in parasitism risk, a Type III functional response leads to uncorrelated same-year host-parasitoid densities. This can be intuitively understood from the fact that for an accelerating attack rate the equilibrium adult host density (19) becomes a decreasing function *R*, while the parasitoid density remains an increasing function of *R*. Our stochastic simulations reveal strong positively correlated densities across successive years resulting in a highly non-monotonic cross-correlation function (Fig. 3). Overall this study highlights the contrasting cross-correlations that emerge from the stochastic dynamics of host-parasitoid interactions providing a valuable tool to infer and discriminate ecological processes. Finally, we also mention several limitations of our work. In order to obtain analytical insights into the impacts of environmental stochasticity, we kept the model dynamics simple by ignoring several population factors, such as parasitoid handing times that lead to Type II functional responses, limited egg capacity of adult parasitoids, interference between parasitoids, density-dependent host/parasitoid mortalities, and host density-dependence of parasitism risk. It will be interesting to see as part of future work how the coupling of these processes with the inherent nonlinear dynamics perturbed by annual fluctuations in parameters shapes the observed population dynamics of host-parasitoid communities.

## Methods

To numerically simulate the stochastic discrete-time model

24a $$\begin{aligned} H_{t+1}= & {} R_t H_tf(R_tH_t,P_t) \end{aligned}$$

24b $$\begin{aligned} P_{t+1}= & {} k R_t H_t\left[ 1-f(R_tH_t,P_t)\right] \end{aligned}$$

we independently draw the random variable $R_t$ in each year *t* from a lognormal distribution with mean *R* and variance $\sigma ^2_R$. The model was simulated in Microsoft Excel using two statistical functions: *RAND* that draws a uniformly distributed number between 0 and 1, and the *NORMINV* function for computing the inverse of the normal cumulative distribution given a specified mean and standard deviation. Thus, the command

25 $$\begin{aligned} NORMINV(RAND(), R, \sigma _R) \end{aligned}$$

can be used to draw a normal random variable with mean *R* and standard deviation $\sigma _R$. To generate a lognormal random variable with mean *R* and variance $\sigma ^2_R$ we use

26 $$\begin{aligned} EXP(NORMINV(RAND(), x, y)), \ \ x=\log R-\frac{\log \left( 1+\frac{\sigma _R^2}{R^2}\right) }{2}, \ \ y=\sqrt{\log \left( 1+\frac{\sigma _R^2}{R^2}\right) } \end{aligned}$$

to draw $R_t$ in each year *t*. For performing the simulations we first determined the mean equilibrium densities by numerically solving the equations

27 $$\begin{aligned} 1=R f(RH^*,P^*), \ \ P^* = (R-1)H^* \end{aligned}$$

in Wolfram Mathematica. These mean equilibrium densities were used as initial conditions. The entire trace overtime was normalized by these initial conditions, and hence the starting point of all simulations in Figs. 1 and 3 is one. The correlations in Fig. 3 were determined using the *CORREL* function in Microsoft Excel. All analytical calculations including solving the Lyapunov equation (12) was done in Wolfram Mathematica.
